# α-l-Glucose

**DOI:** 10.1107/S2414314626008874

**Published:** 2026-08-28

**Authors:** Tomoko Takashita, Yoji Horii, Tomohiko Ishii

**Affiliations:** a Graduate School of Science for Creative Emergence Kagawa University, 2217-20 Hayashi-cho Takamatsu Kagawa 761-0396 Japan; Katholieke Universiteit Leuven, Belgium

**Keywords:** crystal structure, hydrogen bonding, rare sugar, glucose, monosaccharide

## Abstract

Commercially available l-glucose, C_6_H_12_O_6_, was crystallized by slow evaporation from aqueous solution at room temperature affording colorless block-shaped single crystals suitable for single-crystal X-ray diffraction. The title compound crystallizes as α-l-glucose in the ortho­rhom­bic space group *P*2_1_2_1_2_1_, with one mol­ecule in the asymmetric unit and *Z* = 4. In the crystal, the mol­ecules are linked by O—H⋯O hydrogen bonds, forming a three-dimensional hydrogen-bonded network. This study provides an experimentally determined structural dataset for the rare l enanti­omer, which is not currently represented in the Cambridge Structural Database.

## Abstract 2

The title compound, α-l-glucose, was crystallized by slow evaporation from aqueous solution. It crystallizes in the ortho­rhom­bic space group *P*2_1_2_1_2_1_, and the mol­ecules are linked by O—H⋯O hydrogen bonds into a three-dimensional network.

## Graphical abstract

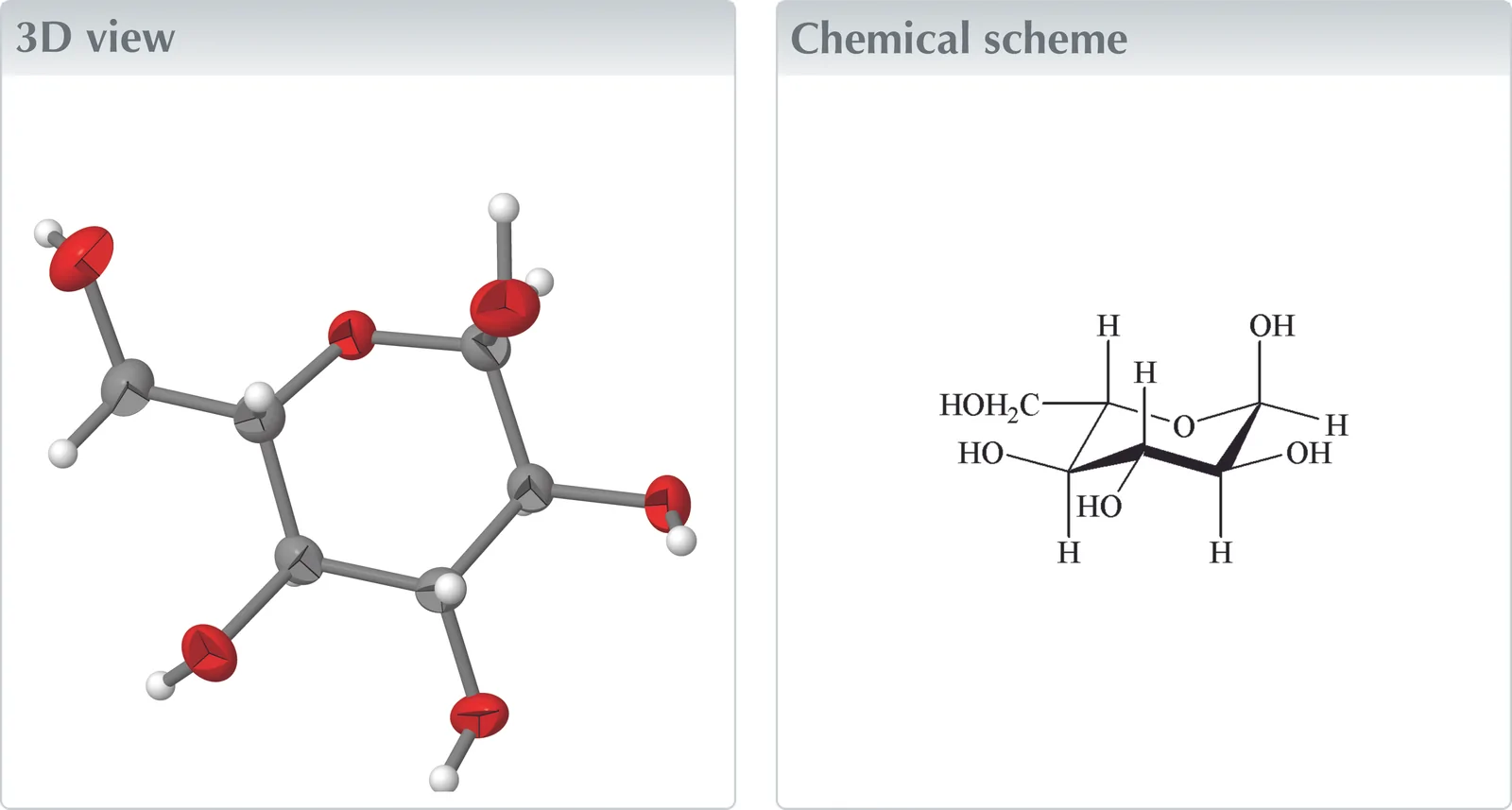

## Structure description

Rare sugars are monosaccharides and derivatives that occur only in limited qu­anti­ties in nature. The development of systematic bioproduction strategies has facilitated access to a wide range of rare hexoses (Izumori, 2002 ▸). l-Glucose is a rare sugar and the enanti­omer of the naturally occurring d-glucose. The Cambridge Structural Database (CSD, version 6.00, update of August 2025; Groom *et al.*, 2016 ▸) contains several crystal structures of α-d-glucose, including the neutron-diffraction structures reported by Brown & Levy (1965 ▸, 1979 ▸; CSD refcodes GLUCSA and GLUCSA10, respectively). In contrast, no experimentally determined crystal structure of α-l-glucose was identified in our CSD search. Although the l enanti­omer is expected to be related to the d enanti­omer by inversion, its direct experimental determination provides an enanti­omer-specific dataset comprising atomic coordinates, mol­ecular conformation and hydrogen-bond geometry. The present structure therefore fills a gap in the crystallographic record and contributes to the systematic accumulation of structural data for rare sugars.

**Figure 1:**
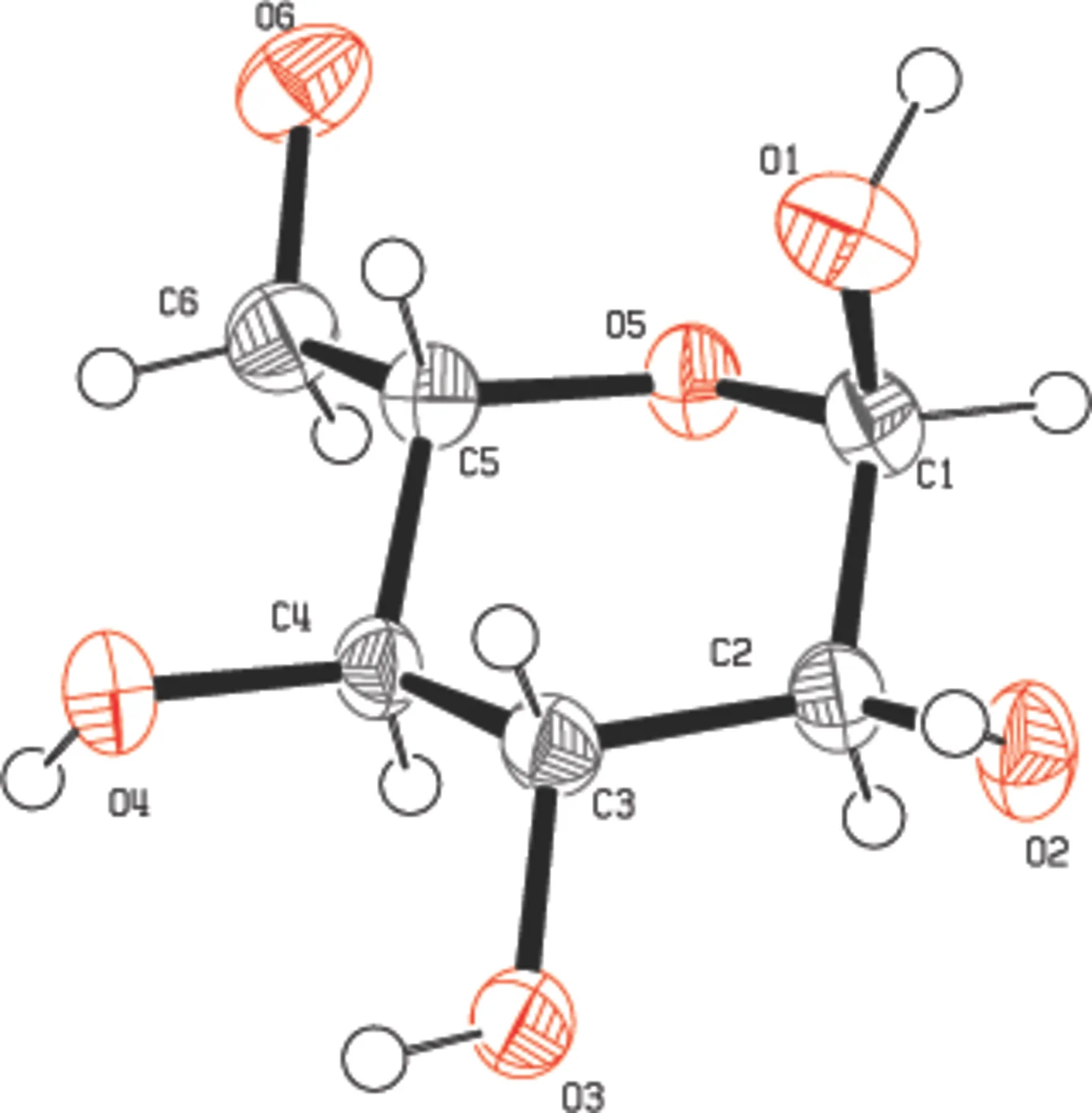
The mol­ecular structure of the title compound, showing the atom-labeling scheme. Displacement ellipsoids are drawn at the 50% probability level. Hydrogen atoms are shown as spheres of arbitrary radius.

The title compound crystallizes in the ortho­rhom­bic space group *P*2_1_2_1_2_1_. The asymmetric unit contains one mol­ecule of α-l-glucose in the pyran­ose form (Fig. 1 ▸). The pyran­ose ring adopts a ^1^*C*_4_ chair conformation, with the anomeric hy­droxy group at C1 occupying an axial position, while the hy­droxy groups at C2, C3 and C4 and the hy­droxy­methyl group at C5 occupy equatorial positions. The Flack parameter of −0.29 (12) was not sufficiently precise to establish the absolute structure independently. The absolute configuration was therefore assigned on the basis of the known identity of the commercially available l-glucose used for crystallization. On this basis, the configurations of the stereogenic centres C1, C2, C3, C4 and C5 are *R*, *S*, *R*, *R* and *S*, respectively. The conformation of the hy­droxy­methyl group is characterized by an O5—C5—C6—O6 torsion angle of −70.7 (3)°. A least-squares fit of the 12 non-hydrogen atoms of the title mol­ecule to those of the α-d-glucose structure reported by Brown & Levy (1965 ▸; CSD refcode GLUCSA) gave r.m.s. deviations of 0.910 Å and 0.013 Å without and with inversion, respectively. The very small r.m.s. deviation obtained after inversion indicates that there is no appreciable difference between the mol­ecular conformations of the two enanti­omers.

**Figure 2:**
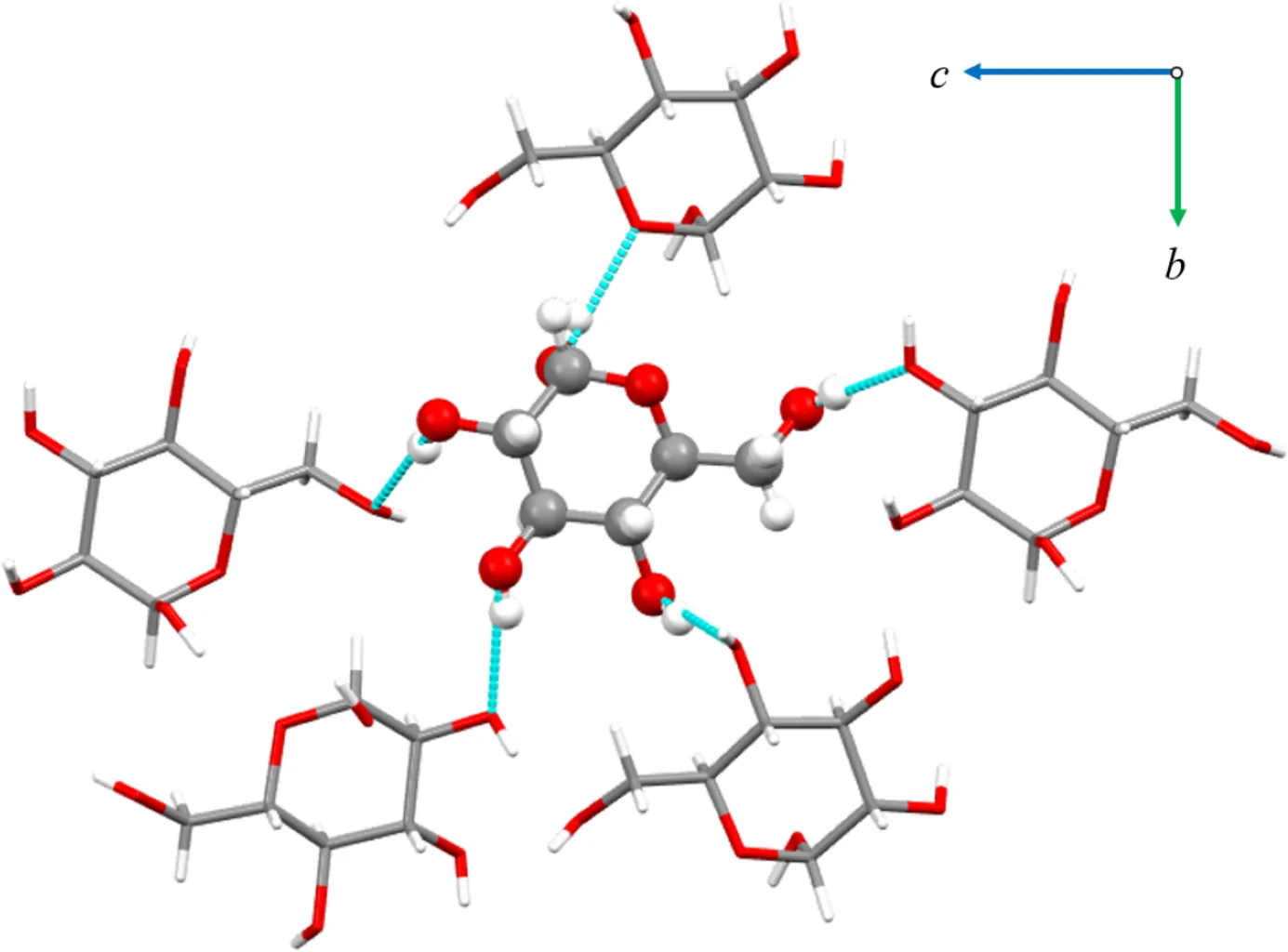
Crystal packing of the title compound viewed along the *a* axis (projection onto the *bc* plane), with the *b* and *c* unit-cell directions indicated. The central glucose mol­ecule is shown using a ball-and-stick representation, whereas the surrounding mol­ecules are shown using a capped-stick representation. The O1—H1⋯O5, O2—H2⋯O6, O3—H3⋯O2, O4—H4⋯O4 and O6—H6⋯O3 hydrogen bonds listed in Table 1 ▸ are shown as dashed lines.

In the crystal, each hy­droxy group acts as a donor in an O—H⋯O hydrogen bond (Table 1 ▸). Collectively, these inter­actions link the mol­ecules into a three-dimensional hydrogen-bonded network, as shown in Fig. 2 ▸.

## Synthesis and crystallization

Commercially available l-glucose (Sigma–Aldrich) was used as received without further purification. The sample was dissolved in water, and the solution was allowed to evaporate slowly at room temperature. Colorless block-shaped single crystals suitable for single-crystal X-ray diffraction were obtained.

## Refinement

Crystal data, data collection and structure refinement details are summarized in Table 2 ▸. The Flack parameter was −0.29 (12), determined using 457 quotients (Parsons *et al.*, 2013 ▸), and did not permit a reliable determination of the absolute structure from the diffraction data alone. The absolute configuration was therefore assigned from the known identity of the commercially available l-glucose used for crystallization.

## Supplementary Material

Crystal structure: contains datablock(s) I. DOI: 10.1107/S2414314626008874/vm4083sup1.cif

Structure factors: contains datablock(s) I. DOI: 10.1107/S2414314626008874/vm4083Isup2.hkl

CCDC reference: 2577717

Additional supporting information:  crystallographic information; 3D view; checkCIF report

## Figures and Tables

**Table 1:** Hydrogen-bond geometry (Å, °)

*D*—H⋯*A*	*D*—H	H⋯*A*	*D*⋯*A*	*D*—H⋯*A*
O1—H1⋯O5^i^	0.95 (4)	1.91 (4)	2.836 (3)	163 (4)
O2—H2⋯O6^ii^	0.90 (4)	1.87 (4)	2.760 (3)	166 (4)
O3—H3⋯O2^iii^	0.83 (4)	1.89 (4)	2.698 (3)	165 (3)
O4—H4⋯O4^iv^	0.85 (5)	1.93 (5)	2.7625 (17)	165 (5)
O6—H6⋯O3^v^	0.84 (4)	1.86 (4)	2.704 (3)	175 (4)

Symmetry codes: (i) 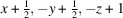; (ii) 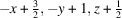; (iii) 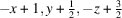; (iv) 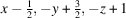; (v) 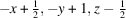.

**Table 2:** Experimental details

Crystal data	
Chemical formula	C_6_H_12_O_6_
*M*_r_	180.16
Crystal system, space group	Orthorhombic, *P*2_1_2_1_2_1_
Temperature (K)	296
*a*, *b*, *c* (Å)	4.9536 (4), 10.3185 (7), 14.7798 (11)
*V* (Å^3^)	755.45 (9)
*Z*	4
Radiation type	Cu *K*α
μ (mm^−1^)	1.26
Crystal size (mm)	0.1 × 0.1 × 0.1

Data collection	
Diffractometer	Rigaku R-AXIS RAPID
Absorption correction	Multi-scan (*ABSCOR*; Rigaku, 1995 ▸)
*T*_min_, *T*_max_	0.656, 1.000
No. of measured, independent and observed [*I* > 2σ(*I*)] reflections	8312, 1374, 1271
*R*_int_	0.043
(sin θ/λ)_max_ (Å^−1^)	0.602

Refinement	
*R*[*F*^2^ > 2σ(*F*^2^)], *wR*(*F*^2^), *S*	0.033, 0.076, 1.06
No. of reflections	1374
No. of parameters	133
H-atom treatment	H atoms treated by a mixture of independent and constrained refinement
Δρ_max_, Δρ_min_ (e Å^−3^)	0.14, −0.16
Absolute structure	Flack *x* determined using 457 quotients [(*I*^+^)−(*I*^−^)]/[(*I*^+^)+(*I*^−^)] (Parsons *et al.*, 2013 ▸)
Absolute structure parameter	−0.29 (12)

Computer programs: *RAPID-AUTO* (Rigaku, 2009 ▸), *SHELXT2018/2* (Sheldrick, 2015*a* ▸), *SHELXL2018/3* (Sheldrick, 2015*b* ▸) and *OLEX2* (Dolomanov *et al.*, 2009 ▸).

## full crystallographic data

### α-L-Glucose Crystal data

**Table:**

C_6_H_12_O_6_	*D*_x_ = 1.584 Mg m^−^^3^
*M**_r_* = 180.16	Cu *K*α radiation, λ = 1.54187 Å
Orthorhombic, *P*2_1_2_1_2_1_	Cell parameters from 7806 reflections
*a* = 4.9536 (4) Å	θ = 3.0–68.4°
*b* = 10.3185 (7) Å	µ = 1.26 mm^−^^1^
*c* = 14.7798 (11) Å	*T* = 296 K
*V* = 755.45 (9) Å^3^	Block, clear light colourless
*Z* = 4	0.1 × 0.1 × 0.1 mm
*F*(000) = 384	

### α-L-Glucose Data collection

**Table:**

Rigaku R-AXIS RAPID diffractometer	1271 reflections with *I* > 2σ(*I*)
Detector resolution: 10.000 pixels mm^-1^	*R*_int_ = 0.043
ω scans	θ_max_ = 68.1°, θ_min_ = 5.2°
Absorption correction: multi-scan (ABSCOR; Rigaku, 1995)	*h* = −5→5
*T*_min_ = 0.656, *T*_max_ = 1.000	*k* = −12→12
8312 measured reflections	*l* = −17→17
1374 independent reflections	

### α-L-Glucose Refinement

**Table:**

Refinement on *F*^2^	Hydrogen site location: mixed
Least-squares matrix: full	H atoms treated by a mixture of independent and constrained refinement
*R*[*F*^2^ > 2σ(*F*^2^)] = 0.033	*w* = 1/[σ^2^(*F*_o_^2^) + (0.0303*P*)^2^ + 0.2146*P*] where *P* = (*F*_o_^2^ + 2*F*_c_^2^)/3
*wR*(*F*^2^) = 0.076	(Δ/σ)_max_ < 0.001
*S* = 1.06	Δρ_max_ = 0.14 e Å^−^^3^
1374 reflections	Δρ_min_ = −0.15 e Å^−^^3^
133 parameters	Absolute structure: Flack *x* determined using 457 quotients [(*I*^+^)-(*I*^-^)]/[(*I*^+^)+(*I*^-^)] (Parsons *et al.*, 2013)
0 restraints	Absolute structure parameter: −0.29 (12)
Primary atom site location: dual	

### α-L-Glucose Special details

**Table:**

Geometry. All esds (except the esd in the dihedral angle between two l.s. planes) are estimated using the full covariance matrix. The cell esds are taken into account individually in the estimation of esds in distances, angles and torsion angles; correlations between esds in cell parameters are only used when they are defined by crystal symmetry. An approximate (isotropic) treatment of cell esds is used for estimating esds involving l.s. planes.
Refinement. All non-hydrogen atoms were refined anisotropically. The hydroxy H atoms H1, H2, H3, H4 and H6 and the C-bound H atom H1A were located in difference-Fourier maps and refined freely. The remaining C-bound H atoms were placed in calculated positions and refined using constrained models.

### α-L-Glucose Fractional atomic coordinates and isotropic or equivalent isotropic displacement parameters (Å^2^)

**Table:**

	*x*	*y*	*z*	*U*_iso_*/*U*_eq_	
O1	0.8530 (4)	0.3471 (2)	0.60082 (14)	0.0418 (6)	
H1	0.918 (10)	0.273 (4)	0.570 (3)	0.087 (14)*	
O2	0.5651 (5)	0.38741 (19)	0.75979 (12)	0.0377 (5)	
H2	0.703 (9)	0.441 (4)	0.774 (3)	0.082 (15)*	
O3	0.3351 (5)	0.6328 (2)	0.71548 (13)	0.0412 (6)	
H3	0.371 (8)	0.711 (4)	0.713 (2)	0.063 (12)*	
O4	0.4329 (5)	0.71765 (18)	0.53468 (13)	0.0344 (5)	
H4	0.289 (9)	0.752 (5)	0.514 (3)	0.085 (16)*	
O5	0.4615 (4)	0.36546 (16)	0.51522 (11)	0.0302 (5)	
O6	0.5544 (5)	0.4462 (2)	0.33123 (14)	0.0454 (6)	
H6	0.437 (8)	0.417 (4)	0.295 (2)	0.058 (12)*	
C1	0.5752 (6)	0.3328 (3)	0.60101 (17)	0.0295 (6)	
H1A	0.526 (4)	0.233 (2)	0.6127 (14)	0.003 (5)*	
C2	0.4535 (6)	0.4187 (2)	0.67401 (16)	0.0287 (6)	
H2A	0.259784	0.399836	0.676647	0.034*	
C3	0.4844 (6)	0.5609 (2)	0.65125 (16)	0.0282 (7)	
H3A	0.675258	0.585394	0.654612	0.034*	
C4	0.3795 (6)	0.5855 (2)	0.55691 (17)	0.0266 (6)	
H4A	0.183949	0.571024	0.556111	0.032*	
C5	0.5113 (6)	0.4965 (2)	0.48833 (16)	0.0282 (6)	
H5	0.706301	0.512517	0.487074	0.034*	
C6	0.3971 (7)	0.5145 (3)	0.39572 (18)	0.0376 (7)	
H6A	0.212706	0.482996	0.394260	0.045*	
H6B	0.395072	0.606009	0.380678	0.045*	

### α-L-Glucose Atomic displacement parameters (Å^2^)

**Table:**

	*U*^11^	*U*^22^	*U*^33^	*U*^12^	*U*^13^	*U*^23^
O1	0.0390 (14)	0.0445 (13)	0.0418 (12)	0.0081 (11)	−0.0048 (10)	−0.0091 (10)
O2	0.0585 (15)	0.0289 (11)	0.0258 (9)	0.0000 (11)	−0.0055 (10)	0.0038 (8)
O3	0.0674 (17)	0.0233 (11)	0.0329 (10)	0.0009 (10)	0.0165 (10)	−0.0026 (8)
O4	0.0400 (13)	0.0247 (10)	0.0385 (11)	−0.0014 (10)	−0.0010 (10)	0.0050 (8)
O5	0.0402 (12)	0.0248 (10)	0.0257 (9)	−0.0023 (9)	−0.0051 (8)	−0.0018 (7)
O6	0.0519 (15)	0.0552 (14)	0.0291 (11)	−0.0086 (13)	0.0052 (11)	−0.0083 (10)
C1	0.0334 (17)	0.0258 (14)	0.0292 (13)	0.0034 (13)	−0.0048 (12)	−0.0006 (11)
C2	0.0356 (17)	0.0241 (14)	0.0264 (13)	0.0001 (13)	−0.0021 (12)	0.0009 (10)
C3	0.0358 (18)	0.0232 (13)	0.0257 (13)	0.0009 (12)	0.0012 (11)	−0.0028 (10)
C4	0.0273 (16)	0.0217 (13)	0.0306 (14)	−0.0023 (11)	0.0008 (11)	0.0028 (10)
C5	0.0288 (17)	0.0274 (14)	0.0285 (13)	−0.0025 (11)	−0.0024 (11)	−0.0008 (10)
C6	0.0444 (19)	0.0399 (17)	0.0284 (14)	0.0009 (15)	−0.0005 (13)	−0.0009 (12)

### α-L-Glucose Geometric parameters (Å, º)

**Table:**

O1—H1	0.95 (4)	C1—H1A	1.07 (2)
O1—C1	1.384 (4)	C1—C2	1.521 (4)
O2—H2	0.90 (4)	C2—H2A	0.9800
O2—C2	1.420 (3)	C2—C3	1.513 (4)
O3—H3	0.83 (4)	C3—H3A	0.9800
O3—C3	1.413 (3)	C3—C4	1.509 (3)
O4—H4	0.85 (5)	C4—H4A	0.9800
O4—C4	1.428 (3)	C4—C5	1.516 (3)
O5—C1	1.428 (3)	C5—H5	0.9800
O5—C5	1.430 (3)	C5—C6	1.493 (3)
O6—H6	0.84 (4)	C6—H6A	0.9700
O6—C6	1.419 (4)	C6—H6B	0.9700
			
C1—O1—H1	105 (3)	C2—C3—H3A	109.6
C2—O2—H2	111 (3)	C4—C3—C2	109.5 (2)
C3—O3—H3	111 (3)	C4—C3—H3A	109.6
C4—O4—H4	109 (3)	O4—C4—C3	108.0 (2)
C1—O5—C5	113.68 (19)	O4—C4—H4A	109.0
C6—O6—H6	103 (3)	O4—C4—C5	110.2 (2)
O1—C1—O5	111.4 (2)	C3—C4—H4A	109.0
O1—C1—H1A	109.2 (12)	C3—C4—C5	111.6 (2)
O1—C1—C2	109.5 (2)	C5—C4—H4A	109.0
O5—C1—H1A	106.2 (11)	O5—C5—C4	108.2 (2)
O5—C1—C2	109.7 (2)	O5—C5—H5	109.6
C2—C1—H1A	110.8 (12)	O5—C5—C6	107.9 (2)
O2—C2—C1	110.3 (2)	C4—C5—H5	109.6
O2—C2—H2A	107.5	C6—C5—C4	112.0 (2)
O2—C2—C3	112.3 (2)	C6—C5—H5	109.6
C1—C2—H2A	107.5	O6—C6—C5	110.2 (3)
C3—C2—C1	111.6 (2)	O6—C6—H6A	109.6
C3—C2—H2A	107.5	O6—C6—H6B	109.6
O3—C3—C2	107.9 (2)	C5—C6—H6A	109.6
O3—C3—H3A	109.6	C5—C6—H6B	109.6
O3—C3—C4	110.6 (2)	H6A—C6—H6B	108.1
			
O1—C1—C2—O2	−57.4 (3)	C1—O5—C5—C4	−62.1 (3)
O1—C1—C2—C3	68.1 (3)	C1—O5—C5—C6	176.5 (2)
O2—C2—C3—O3	−63.5 (3)	C1—C2—C3—O3	172.1 (2)
O2—C2—C3—C4	176.1 (2)	C1—C2—C3—C4	51.7 (3)
O3—C3—C4—O4	66.2 (3)	C2—C3—C4—O4	−175.0 (2)
O3—C3—C4—C5	−172.5 (2)	C2—C3—C4—C5	−53.8 (3)
O4—C4—C5—O5	177.8 (2)	C3—C4—C5—O5	57.8 (3)
O4—C4—C5—C6	−63.4 (3)	C3—C4—C5—C6	176.6 (2)
O5—C1—C2—O2	−179.9 (2)	C4—C5—C6—O6	170.3 (2)
O5—C1—C2—C3	−54.4 (3)	C5—O5—C1—O1	−60.5 (3)
O5—C5—C6—O6	−70.7 (3)	C5—O5—C1—C2	60.8 (3)

### α-L-Glucose Hydrogen-bond geometry (Å, º)

**Table:**

*D*—H···*A*	*D*—H	H···*A*	*D*···*A*	*D*—H···*A*
O1—H1···O5^i^	0.95 (4)	1.91 (4)	2.836 (3)	163 (4)
O2—H2···O6^ii^	0.90 (4)	1.87 (4)	2.760 (3)	166 (4)
O3—H3···O2^iii^	0.83 (4)	1.89 (4)	2.698 (3)	165 (3)
O4—H4···O4^iv^	0.85 (5)	1.93 (5)	2.7625 (17)	165 (5)
O6—H6···O3^v^	0.84 (4)	1.86 (4)	2.704 (3)	175 (4)

Symmetry codes: (i) *x*+1/2, −*y*+1/2, −*z*+1; (ii) −*x*+3/2, −*y*+1, *z*+1/2; (iii) −*x*+1, *y*+1/2, −*z*+3/2; (iv) *x*−1/2, −*y*+3/2, −*z*+1; (v) −*x*+1/2, −*y*+1, *z*−1/2.

## References

[bb1] Brown, G. M. & Levy, H. A. (1965). *Science* **147**, 1038–1039. 10.1126/science.147.3661.1038-a 14245779

[bb2] Brown, G. M. & Levy, H. A. (1979). *Acta Cryst.* B**35**, 656–659.

[bb3] Dolomanov, O. V., Bourhis, L. J., Gildea, R. J., Howard, J. A. K. & Puschmann, H. (2009). *J. Appl. Cryst.* **42**, 339–341.

[bb4] Groom, C. R., Bruno, I. J., Lightfoot, M. P. & Ward, S. C. (2016). *Acta Cryst.* B**72**, 171–179. 10.1107/S2052520616003954 PMC4822653 27048719

[bb5] Izumori, K. (2002). *Naturwissenschaften* **89**, 120–124. 10.1007/s00114-002-0297-z 12046631

[bb6] Parsons, S., Flack, H. D. & Wagner, T. (2013). *Acta Cryst.* B**69**, 249–259. 10.1107/S2052519213010014 PMC3661305 23719469

[bb7] Rigaku (1995). *ABSCOR*. Rigaku Corporation, Tokyo, Japan.

[bb8] Rigaku (2009). RAPID AUTO. Rigaku Corporation, Tokyo, Japan.

[bb9] Sheldrick, G. M. (2015*a*). *Acta Cryst.* A**71**, 3–8.

[bb10] Sheldrick, G. M. (2015*b*). *Acta Cryst.* C**71**, 3–8.

